# Properties of the ternary complex formed by yeast eIF4E, p20 and mRNA

**DOI:** 10.1038/s41598-018-25273-3

**Published:** 2018-04-30

**Authors:** Nick Arndt, Daniela Ross-Kaschitza, Artyom Kojukhov, Anton A. Komar, Michael Altmann

**Affiliations:** 1 Institut für Biochemie und Molekulare Medizin, Bühlstr. 28, 3012 Bern, Switzerland; 2 Center for Gene Regulation in Health and Disease and Department of Biological, Geological and Environmental Sciences, Cleveland State University, Cleveland, OH 44115 USA

## Abstract

Yeast p20 is a small, acidic protein that binds eIF4E, the cap-binding protein. It has been proposed to affect mRNA translation and degradation, however p20′s function as an eIF4E-binding protein (4E-BP) and its physiological significance has not been clearly established. In this paper we present data demonstrating that p20 is capable of binding directly to mRNA due to electrostatic interaction of a stretch of arginine and histidine residues in the protein with negatively charged phosphates in the mRNA backbone. This interaction contributes to formation of a ternary eIF4E/p20/capped mRNA complex that is more stable than complexes composed of capped mRNA bound to eIF4E in the absence of p20. eIF4E/p20 complex was found to have a more pronounced stimulatory effect on capped mRNA translation than purified eIF4E alone. Addition of peptides containing the eIF4E-binding domains present in p20 (motif  YTIDELF), in eIF4G (motif  YGPTFLL) or Eap1 (motif  YSMNELY) completely inhibited eIF4E-dependent capped mRNA translation (*in vitro*), but had a greatly reduced inhibitory effect when eIF4E/p20 complex was present. We propose that the eIF4E/p20/mRNA complex serves as a stable depository of mRNAs existing in a dynamic equilibrium with other complexes such as eIF4E/eIF4G (required for translation) and eIF4E/Eap1 (required for mRNA degradation).

## Introduction

Four different proteins have been shown to interact with the mRNA cap-binding protein eIF4E (eukaryotic translation initiation factor 4E) in *Saccharomyces cerevisiae*. These eIF4E binding proteins (BPs) are Eap1^1^, p20^2^, and two isoforms of translation initiation factor eIF4G, eIF4G1 and eIF4G2^3^. The small acidic p20 protein (encoded by the non-essential *CAF20* gene) co-purifies with yeast eIF4E on m^7^GDP-Sepharose and was initially described as a repressor of cap-dependent translation^2,4^. Deletion of p20 was shown to alleviate slow-growth and cold-sensitivity phenotypes caused by mutations in translation initiation factors such as eIF4B, eIF4E or eIF4G1, while its overexpression had the opposite effect of enhancing these phenotypes^5^. p20 also appears to play a role in mRNA decay as evidenced by the finding that its deletion suppresses phenotypes caused by deletion of genes encoding two factors involved in mRNA degradation, the decapping factor Pat1p and the DEAD box helicase Dhh1p^6^.

Biochemical, structural and *in silico* analyses of 4E-BPs and eIF4Gs from various metazoan sources identified a conserved eIF4E-binding motif consisting of Tyr (X)_4_ Leu-φ, with X being any amino acid and φ being a hydrophobic amino acid. This sequence folds into an alpha-helical conformation and binds to the dorsal surface of eIF4E, distal to the mRNA cap-binding site^7,8^. This interaction enhances the affinity of eIF4E for the cap-structure^9,10^. Recently, metazoan 4E-BP and eIF4G and plant eIF4G peptide crystal structures revealed additional non-canonical binding motifs that consist of hydrophobic amino acids and interact with the lateral surface of eIF4E at sites flanking the canonical binding site^11–15^. 4E-BPs seem to have a competitive advantage over eIF4G due to higher stability of the complexes they form with eIF4E attributed to a less flexible linker region positioned between canonical and non-canonical binding motifs^14^. Binding of metazoan 4E-BPs to eIF4E is regulated by ordered phosphorylation at several sites^16–18^. Hypophosphorylated 4E-BP binds tightly to eIF4E which prevents translation initiation. Phosphorylation of two threonine residues upstream of the canonical eIF4E binding motif (Thr37 and Thr46) in 4E-BP’s linker region leads to a conformational change that serves as a priming event allowing for further phosphorylation of serine and threonine residues followed by displacement of 4E-BP from eIF4E complexes by eIF4G^13,17,18^.

p20 only exists in yeast species of the subphylum *Saccharomycotina*^19^. While it is similar to metazoan 4E-BPs in terms of size and presence of a canonical eIF4E-binding motif, yeast p20 does not share any primary sequence homology with metazoan 4E-BPs. Since p20 is subject to phosphorylation in yeast, it has been assumed to be regulated similarly to mammalian 4E-BPs^20^. However, non-phosphorylatable p20 mutants do not display any phenotypic differences compared to cells with wild type p20 (our unpublished data; not shown). The yeast proteins p20, eIF4G1/2 and Eap1 all contain the canonical binding motif and compete for binding to eIF4E. Unlike metazoan 4E-BPs, the binding motif in p20 is located at its N-terminus, not in the middle of the protein (see Supplemental Fig. S1a,b). Structural “embracing” of eIF4E by metazoan and plant 4E-BPs has been described^10,15,21^. Whether a second, non-canonical eIF4E-binding motif^22–26^ is present in p20 remains unknown since a crystal structure of yeast p20 bound to eIF4E has not been reported to date.

Microarray analysis of polysome-associated mRNAs obtained from a p20 knockout yeast strain revealed both translational up- and down-regulation of hundreds of genes, thus suggesting that p20 is not a general repressor of translation, but rather has a more complex regulatory role^22^. Analysis of these data suggested that yeast 4E-BPs bind directly to mRNAs, with particularly high affinity for those possessing long 5′UTRs with pronounced secondary structures and higher negative charge density due to helical organization of negatively charged phosphate residues^23^.

The current study was undertaken to gain further insight into the details of how p20 interacts with eIF4E and mRNA in order to clarify its role in regulating translation. Using MicroScale Thermophoresis^TM^ (MST) and Electrophoretic Mobility Shift Assays (EMSA), purified recombinant proteins, and/or synthetic and *in vitro* transcribed RNAs, we determined dissociation constants for binding of capped or uncapped RNAs to eIF4E alone or complexed with p20. Our results show that p20 interacts directly with mRNA and define a positively charged region of the protein that mediates this interaction, yet is dispensable for binding of p20 to eIF4E. Furthermore, we found that eIF4E/p20 complex binds more strongly to capped mRNA than eIF4E alone and has a greater stimulatory effect on *in vitro* translation of capped mRNAs. In contrast, eIF4E/p20 complex inhibited translation of uncapped mRNAs. Peptides containing eIF4E-binding domains from yeast p20, eIF4G or Eap1 strongly inhibited eIF4E-dependent translation of capped mRNAs, but had a greatly reduced effect in the presence of eIF4E/p20 complex. Taken together, these data argue against p20 acting exclusively as an inhibitor of translation. Rather, the protein plays a dual role, inhibiting translation of uncapped RNAs while enhancing translation of capped RNAs through formation of a stable eIF4E/p20/mRNA ternary complex.

## Results

### p20 binds directly to RNA

In order to investigate interactions between yeast eIF4E, p20 and RNA, we used purified recombinant His-tagged proteins produced in *E. coli*. Codon usage for the two proteins was optimized for expression in *E. coli* using a reverse gene optimization approach developed earlier^24,25^ (see Supplemental Fig. S1c,d). When expressed alone in *E. coli*, p20 was produced at high levels but could not be efficiently purified via its N-terminal His6x-tag due to rapid degradation of the protein (not shown). However, it was possible to efficiently purify the yeast eIF4E/p20 complex from *E. coli* cells co-expressing untagged eIF4E and His-tagged p20, with yields of 1–2 mg of highly purified complex per 1 L culture (Supplemental Fig. S2a). This suggests that co-expression of eIF4E and p20 contributes to proper folding and intracellular stability of p20.

**Figure 1:**
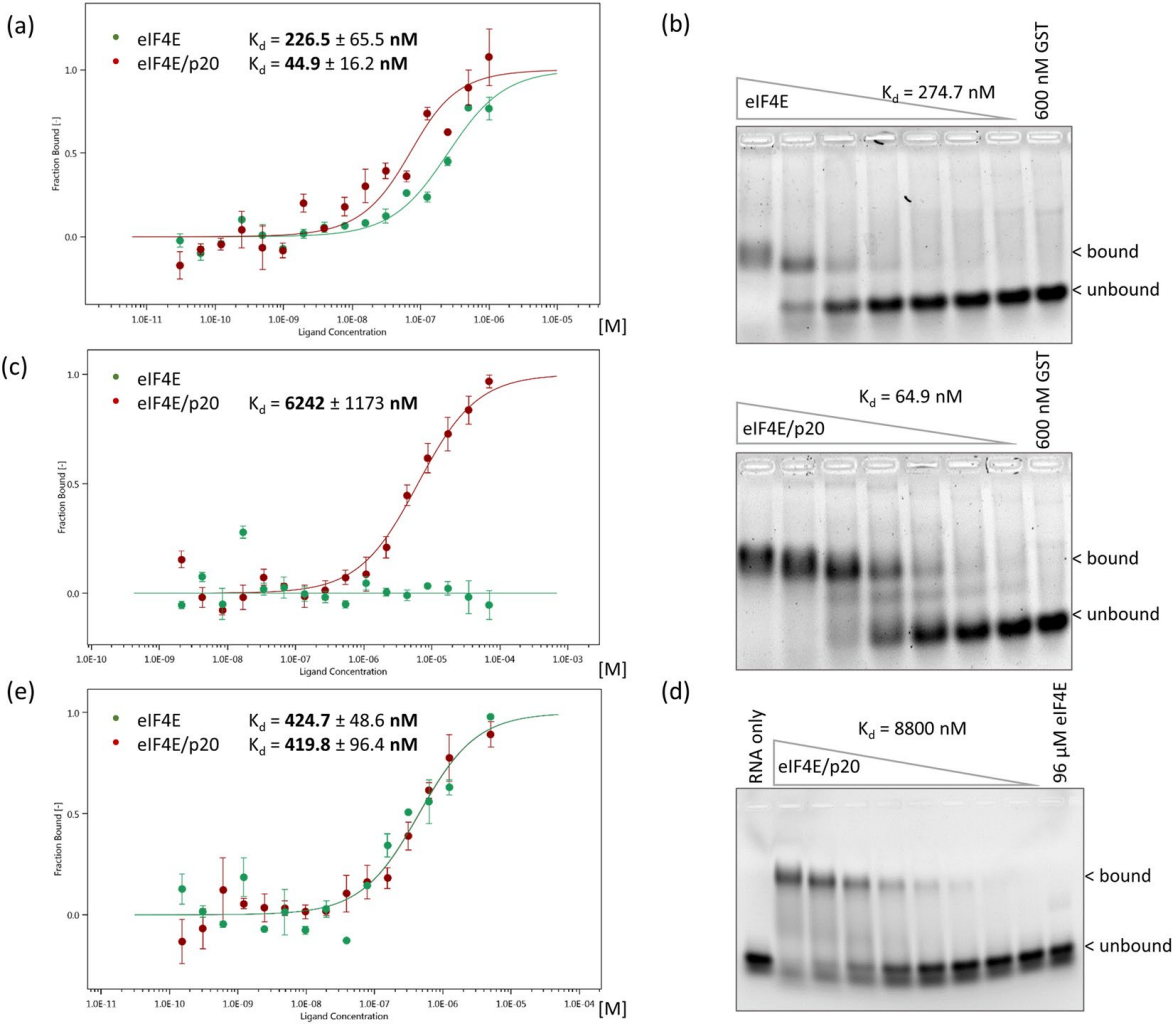
Determination of dissociation constants (K_d_) for eIF4E/p20 complex or eIF4E bound to capped or uncapped RNA by MicroScale Thermophoresis (MST) and Electrophoretic Mobility Shift Assay (EMSA). (**a**) MST: 50 nM RED fluorescence dye-labeled His6x-eIF4E (green) or eIF4E/His6x-p20 complex (red) with serially diluted m^7^GpppG-capped 64 nt long RNA (40 nt of SSA1 5′UTR), starting at 1 µM. Error bars indicate s.d. (n = 3). (**b**) EMSA: 600–0 nM His6x-eIF4E or 600–0 nM eIF4E/His6x-p20 complex with 0.25 µM m^7^GpppG-capped 64 nt long RNA (40 nt of SSA1 5′UTR) probe. SYBR Gold Nucleic Acid Gel Stain. GST (Glutathione-S Transferase) serves as a negative control. (**c**) MST: Serially diluted His6x-eIF4E (green) or eIF4E/His6x-p20 complex (red), starting at 96 µM, with 50 nM 3′FAM-labeled uncapped 40 nt long 5′UTR of SSA1 RNA. Error bars indicate s.d. (n = 3). (**d**) EMSA: 96–0 µM eIF4E/His6x-p20 complex with 1 µM 3′FAM-labeled uncapped 40 nt long RNA probe (5′UTR of SSA1). (**e**) MST: 50 nM RED fluorescence dye-labeled His6x-eIF4E (green) or eIF4E/His6x-p20 complex (red) with serial dilutions of m^7^GpppG starting at 5 µM. Error bars indicate s.d. (n = 3).

The RNA-binding properties of the purified eIF4E/p20 complex and purified eIF4E were compared using two different procedures: MicroScale Thermophoresis^TM^ (MST) and Electrophoretic Mobility Shift Assays (EMSA). We analyzed binding to (i) a synthetic 3′fluorescent-labeled uncapped RNA corresponding to 40 nucleotides of the 5′UTR of SSA1 and (ii) to an *in vitro* transcribed 64 nucleotide long capped RNA consisting of 24 synthetic nucleotides and the same 40 nucleotides of the SSA1 5′UTR (Supplemental Fig. S1e). We found that eIF4E bound quite efficiently to capped RNA (Fig. 1a,b, Table 1), with an approximate K_d_ value of 227 nM (MST) to 275 nM (EMSA) which is comparable to the K_d_ of around 100 nM determined for yeast eIF4E by fluorescence-based gel-shift assays^26^ and for human eIF4E by stopped-flow fluorescence^27^. Interestingly, the eIF4E/p20 complex showed 4- to 5-fold greater affinity for capped RNA, with K_d_ values of 45 nM (MST; Fig. 1a) to 65 nM (EMSA; Fig. 1b, Table 1). This clearly indicates that the affinity of eIF4E for capped mRNAs is considerably enhanced by the presence of its partner p20. Of note, the K_d_ values that we measured for eIF4E/p20/capped mRNA complexes are one order of magnitude higher than those obtained by surface plasmon resonance for human 4E-BP1 or 4E-BP2 bound to m^7^GTP/eIF4E (K_d_ = 2–7 nM)^28,29^.

**Table 1:** Summary of all measured K_d_s (nM).

	Capped mRNA		Uncapped mRNA		Cap-analog
	MST	EMSA	MST	EMSA	MST
eIF4E	226.5	274.7	—	—	424.7
eIF4E/p20	44.9	64.9	6,242	8,800	419.8
eIF4E/p20 6×	423.9	285.1	35,481	—	n.d.

We next tested binding to the 40 nt uncapped SSA1 RNA and, as expected, found that eIF4E alone did not bind at all to the RNA (Fig. 1c for MST, Fig. 1d last lane for EMSA). In contrast, the eIF4E/p20 complex did bind to the uncapped RNA, albeit with rather low affinity [K_d_ = 6.3 µM by MST (Fig. 1c), 8.8 µM by EMSA (Fig. 1d)]. Thus, binding of the complex to uncapped RNA was ~125-150-fold less efficient than to capped RNA. Nevertheless, for both capped and uncapped RNA, eIF4E/p20 RNA-binding was stronger than that of eIF4E alone. Similar experiments could not be performed with p20 alone due to the problems with its purification mentioned above.

We also used EMSA and MST to determine dissociation constants for interaction between eIF4E or eIF4E/p20 complex and the cap-analog m^7^GpppG. This showed no binding preference for either eIF4E or eIF4E/p20 (K_d_ = 420–425 nM for both; Fig. 1e, Table 1). Thus, interaction with the cap- structure appears to be solely due to contacts with eIF4E (presence of p20 does not have any effect), while stabilization of eIF4E/p20/capped mRNA ternary complexes requires longer nucleotide stretches.

Based on these results, we conclude that the eIF4E/p20 complex binds capped mRNA to form a stable ternary complex via interaction of eIF4E with the cap-structure and interaction of p20 with the RNA.

The impact of RNA length and/or sequence on the enhanced RNA-binding properties of the eIF4E/p20 complex (such as the reported preferential binding of yeast eIF4G to RNAs with oligo(U) stretches^30^) remains unclear at this point and will require further investigation.

### The positively charged 3R/3H motif in p20 contributes to its RNA-binding capacity

**Figure 2:**
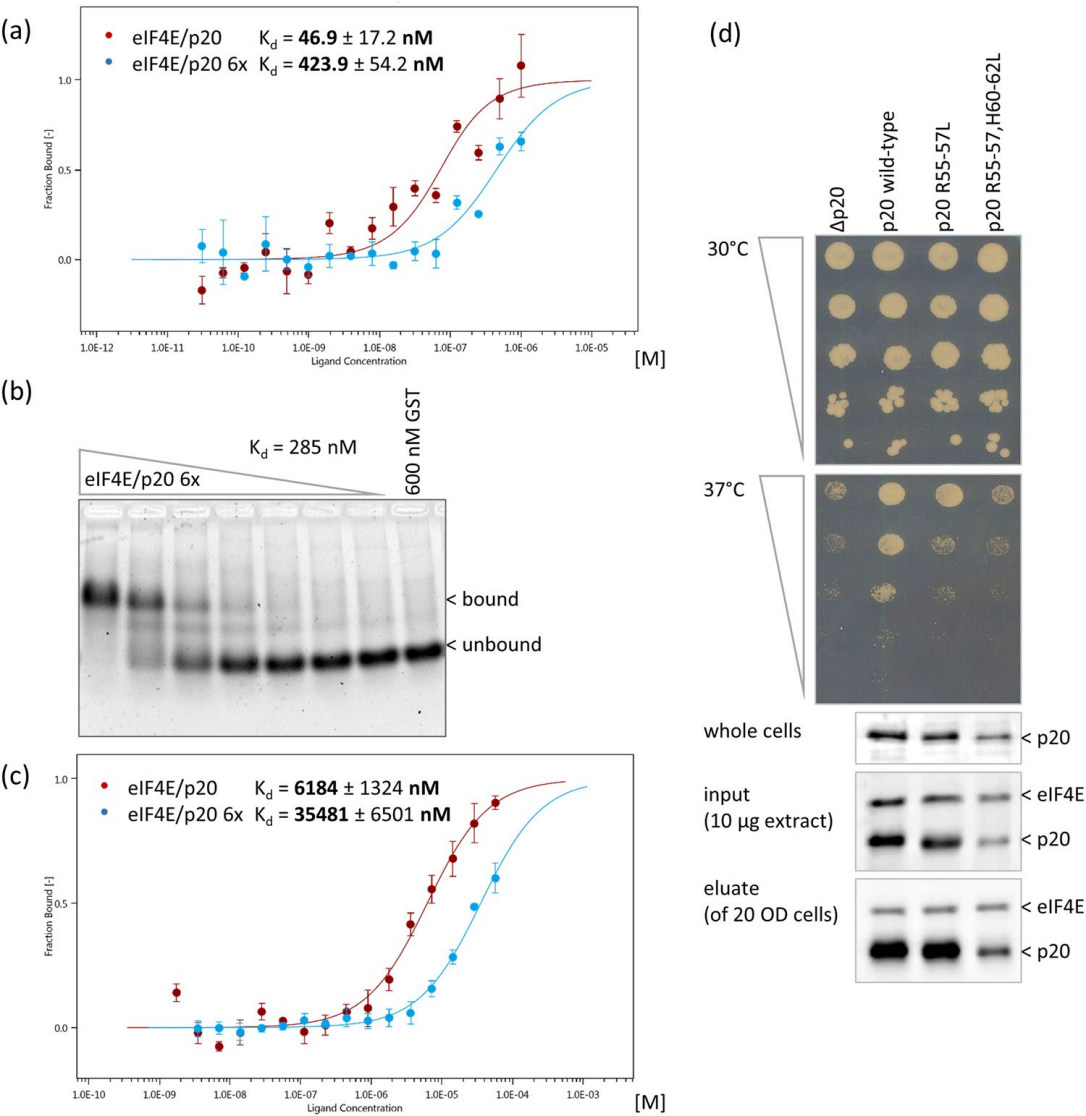
Determination of dissociation constants (K_d_) for eIF4E/p20 wild type complex versus eIF4E/p20 R55-57L, H60-62L (6×) mutant complex bound to capped or uncapped RNA by MicroScale Thermophoresis (MST) and Electrophoretic Mobility Shift Assay (EMSA). (**a**) MST: 50 nM RED fluorescence dye-labeled eIF4E/His6x-p20 complex (red) or eIF4E/His6x-p20 R55-57L, H60-62L (6×) mutant complex (blue) with serially diluted m^7^GpppG-capped 64 nt long RNA (40 nt of SSA1 5′UTR), starting at 1 µM. Error bars indicate s.d. (n = 3). (**b**) EMSA: 600–0 nM His6x-p20 6× mutant/eIF4E complex with 0.25 µM m^7^GpppG-capped 64 nt long RNA probe (40 nt of SSA1 5′UTR). SYBR Gold Nucleic Acid Gel Stain. GST serves as a negative control. (**c**) MST: Serially diluted 96 µM eIF4E/His6x-p20 complex (green) or His6x-p20 6× mutant/eIF4E complex (blue) with 50 nM 3′FAM-labeled uncapped 40 nt long 5′UTR of SSA1 RNA. Error bars indicate s.d. (n = 3). (**d**) Serial dilution of yeast cells carrying p20 knockout (RH2585 ∆p20) or expressing p20 wild type, R55-57L (3×) or R55-57L, H60-62L (6×) mutants. Plates were incubated at 30° or 37 °C for 3 days. Western Blots of whole cells (1/2 OD_600_), “input” extracts used for m^7^GDP-Sepharose pulldown, and eluates are shown. Full-length blots are presented in Supplemental Fig. S3. Blots were stained with polyclonal antibodies against eIF4E or p20 as indicated.

As mentioned in the Introduction, it has been suggested that the similar arrangements of negatively and positively charged amino acids present in yeast eIF4E-binding proteins such as eIF4G1/2, Eap1 and p20 contribute to their RNA-binding capacity^23^. In the case of p20, a motif consisting of three arginines (R55-57) and three histidines (H60-62) separated by two serines (S58, 59; see Supplemental Fig. S1a) might determine the protein’s RNA-binding properties. To test this, we produced mutant recombinant p20 proteins in which either the 3 arginines or all 6 positively charged residues (3 arginines and 3 histidines) were replaced by leucine residues (referred to as “R55-57L” or “3×” and “R55-57, H60-62L” or “6×” mutants, respectively). These mutations had no effect on interaction of p20 with eIF4E (Fig. 2d) and the mutant proteins were efficiently purified from *E. coli* similarly to wild type (WT) p20 (Supplemental Fig. S2a).

When tested in MST and EMSA experiments, the eIF4E/p20 6× mutant complex displayed 9- to 10-fold lower binding affinity for capped RNA compared to the eIF4E/p20 WT complex (Fig. 2a,b). Not surprisingly, the K_d_ values for binding of capped RNA to the eIF4E/p20 6× mutant (K_d_ = 285 nM; Fig. 2b) and to eIF4E alone (K_d_ = 275 nM; Fig. 1b, Table 1) were similar. Together, these results indicate that the presence of the 3 arginine and 3 histidine residues in p20 are important for its RNA-binding capacity. Consistent with this conclusion, analysis of binding to uncapped RNA also showed significantly lower affinity of the eIF4E/p20 6× mutant complex (K_d_ = 35.5 µM) compared to the eIF4E/p20 WT complex (K_d_ = 6.2 µM; Fig. 2c). These results support the hypothesis that binding of p20 to mRNA is mediated by electrostatic interaction of positively charged amino acids in p20 with negatively charged phosphates in the mRNA^23^.

To examine the effect of mutations in the positively charged 3R/3H motif *in vivo*, we constructed pseudohyphenating yeast strains (derivatives of filamentous strain Σ1278b) expressing the 3× and 6× mutant versions of p20 from genomically integrated constructs. Growth of these strains was compared to that of isogenic p20 knockout or p20 WT strains^31^. As opposed to non-hyphenating strains used in previous studies (such as BY4741), knockout of p20 in our pseudohyphenating strain results in a discrete ts- (temperature sensitive) phenotype in which growth is observed at 30 °C but not at 37 °C. This ts-phenotype was reversed by genomic insertion of the gene encoding wild type p20 (Fig. 2d). In contrast, the p20 R55-57L (3×) mutation, and to an even greater extent, the p20 R55-57L, H60-62L (6×) mutation inhibited growth at 37 °C similarly to the p20 knockout. While these results seem to indicate that the 3R/3H motif is critical for p20′s cellular function, Western blotting with antibodies against p20 showed that the 3× mutant and especially the 6× mutant were expressed at lower levels than wild type p20 in the strains used in this study (e.g., protein levels for the 6× mutant were ~30% of p20 WT levels). Therefore, although both the 3× and 6× mutant forms of p20 retain the capacity to bind to eIF4E on a m^7^GDP cap-column (Fig. 2d, lowest panel), we cannot exclude the possibility that reduced growth of cells expressing these mutants at 37 °C is at least partially caused by lower p20 expression.

### The eIF4E/p20 complex enhances translation efficiency *in vitro* in a factor-dependent lysate

**Figure 3:**
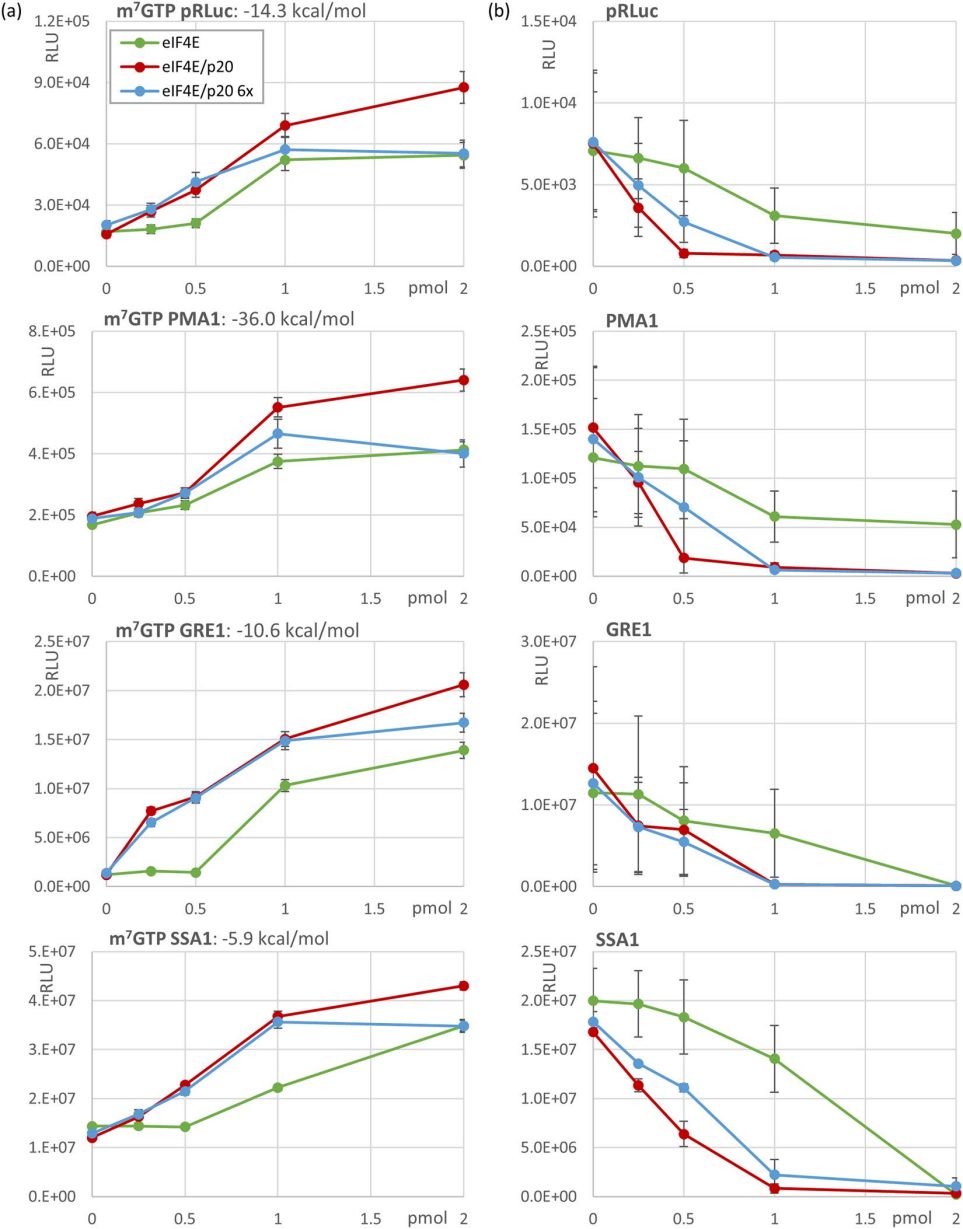
*In vitro* translation assays performed with 10 ng/assay capped or uncapped “average synthetic” RLuc, PMA1, GRE1 or SSA1 5′UTRs fused to Renilla Luciferase ORF and increasing amounts of His6x-eIF4E alone (green), eIF4E/His6x-p20 complex (red) or eIF4E/His6x-p20 6× mutant complex (blue). Data presented are the mean luminescence activity in relative light units (RLU ± s.e.m.; n = 3). The calculated free energy (kcal/mol) of each 5′UTR is shown above each panel in (**a**). (**a**) *In vitro* translation assays of capped RNAs. (**b**) *In vitro* translation assays of uncapped RNAs.

Given the ability of p20 to directly bind RNA and mediate formation of stable eIF4E/p20/mRNA complexes, we were interested in comparing the effects of eIF4E/p20 complex versus eIF4E alone on translation in a controlled *in vitro* system. For this purpose, we prepared translational extracts from a yeast strain carrying an eIF4E ts4-2 mutation combined with a p20 knockout. As previously described, this mutant has a ts-phenotype (no growth at 37 °C) due to the eIF4E ts4-2 mutation^32^. We compared translation of capped Renilla luciferase mRNA constructs carrying different 5′UTRs (for more details see Supplemental Fig. S1e): (i) an “average” synthetic 5′UTR (pRLuc, 41 nt long, predicted ∆G = −14.3 kcal mol^−1^), (ii) the long and structured 5′UTR of PMA1 (289 nt long, predicted ∆G = −36 kcal mol^−1^), (iii) the 5′UTR of GRE1 (77 nt long, predicted ∆G = −10.6 kcal mol^−1^), and (iv) the 5′UTR of SSA1 (69 nt long, predicted ∆G = −5.9 kcal mol^−1^). All four mRNAs contained polyA tails. Addition of purified eIF4E to *in vitro* translation reactions stimulated translation of capped Renilla luciferase mRNAs in a dose-dependent manner regardless of which of the four tested 5′UTR sequences was present (Fig. 3a). Surprisingly, addition of eIF4E/p20 complex resulted in even greater enhancement of translation than addition of eIF4E alone (Fig. 3a). This clearly argues against p20 acting as an inhibitor of translation. Moreover, contrary to expectations, addition of eIF4E/p20 6× mutant protein complex enhanced translation almost as well as eIF4E/p20 WT complex (Fig. 3a). Thus, disruption of p20′s interaction with mRNA (as shown for the 3× and 6× mutants above) does not affect its ability to stimulate eIF4E-dependent translation. It should be noted that while both eIF4E alone and eIF4E/p20 (6× or WT) complex stimulated translation of all four tested reporter mRNAs, the effect was particularly strong for the reporter mRNA containing the GRE1 5′UTR. Whether this observation is significant in terms of the impact of 5′UTR sequence or structure on eIF4E/p20 regulated translation remains unclear at this time.

When the effect of purified eIF4E and p20 on translation of uncapped Renilla luciferase reporter mRNAs containing the four different 5′UTRs was evaluated, we found that eIF4E strongly inhibited translation of all four mRNAs in a dose-dependent manner (Fig. 3b). This was expected since it is known that translation of uncapped mRNA requires active eIF4G which is titered out by increasing amounts of added eIF4E^33^. Surprisingly, however, the inhibitory effect on translation of uncapped mRNAs was even more pronounced when eIF4E/p20 WT or eIF4E/p20 6× mutant complexes were added to reactions rather than eIF4E alone (Fig. 3b). This effect was not due to reduced mRNA stability during incubation in the translational extract (determined by semiquantitive RT-PCR; data not shown). Thus, we conclude that eIF4E/p20 complex – even when p20′s mRNA binding capacity is reduced as in the case of the p20 6× mutant – contributes to inhibition of uncapped mRNA translation. We speculate that this could represent a physiological mechanism to help impede translation of uncapped mRNAs in the process of degradation.

### eIF4E-binding peptides inhibit eIF4E-dependent but not eIF4E/p20-dependent translation

**Figure 4:**
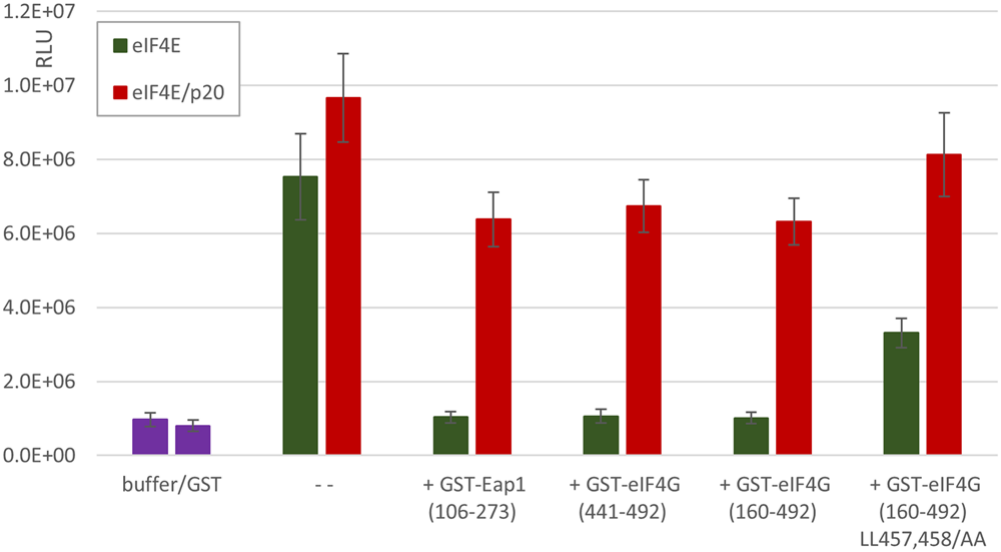
Inhibition of eIF4E-dependent *in vitro* translation by 4E-binding peptides. 1 pmol eIF4E (green) or 1 pmol eIF4E/p20 (red) was added to *in vitro* translation reactions containing 5 ng capped GRE1-Luc mRNA. Additionally, 1 pmol of the following purified GST-fusion proteins were added: GST-Eap1_106–273_, GST-eIF4G_441–492_, GST-eIF4G_160–492_ and GST-eIF4G_160–492_ LL457,458/AA. 1 pmol GST alone had no stimulatory effect on translation. Data presented are the mean luminescence activity in relative light units (RLU ± s.e.m.; n = 3).

In previous work, we showed that peptides containing the eIF4E-binding domain of yeast eIF4G were outcompeted by p20 for interaction with eIF4E^4^. Here, we tested whether exogenously added eIF4E or eIF4E/p20 complex could be outcompeted during *in vitro* translation of capped mRNA by peptides containing eIF4E-binding domains of eIF4G, Eap1 or p20 (Supplemental Fig. S1b). Indeed, stimulation of capped mRNA translation by addition of 1 pmol eIF4E was completely abolished by the presence of equimolar amounts of such peptides (GST-eIF4G_160–492_, GST-eIF4G_441–492_ or GST-Eap1_106–273_; Fig. 4). However, even excess addition (10×) of 4E-binding peptides did not completely inhibit translation (not shown). As a control, we used GST-eIF4G_160–492_ LL459,460/AA which contains two mutations in the canonical eIF4E-binding motif (Supplemental Fig. S1b). This eIF4G mutant was previously shown to cause a ts-phenotype in yeast due to its weakened interaction with eIF4E^33^. Addition of 1 pmol GST-eIF4G_160–492_ LL459,460/AA to our *in vitro* translation system had a less severe impact on eIF4E-dependent translation than GST-eIF4G_160–492_ (Fig. 4).

In similar experiments performed with addition of eIF4E/p20 complex to *in vitro* translation reactions, equimolar amounts of GST-eIF4G_160–492_, GST-eIF4G_441–492_ or GST-Eap1_106–273_ had a much smaller inhibitory effect (Fig. 4). Additionally, GST-eIF4G_160–492_ LL459,460/AA had a minor competitive effect on translation in the presence of eIF4E/p20 complex. Based on these data, we propose that eIF4E and p20 form a stable complex that is not easily disrupted by competition of eIF4E-binding peptides derived from eIF4G or Eap1.

### An N-terminal 18aa p20 peptide inhibits capped mRNA translation but does not affect eIF4E/capped mRNA interaction

**Figure 5:**
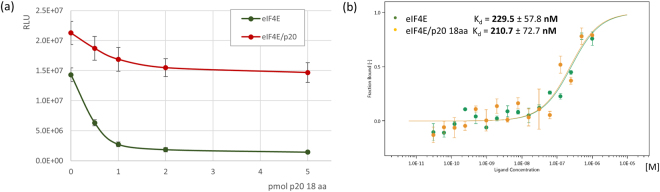
Effect of the N-terminal 18aa p20 peptide on translation and eIF4E binding of capped RNA. (**a**) Increasing amounts of the 18aa N-terminal p20 peptide (up to 5 pmol) were added to *in vitro* translational assays in the presence of 1 pmol eIF4E (green) or 1 pmol eIF4E/p20 (red). Translational assays were performed as described for Fig. 4. (**b**) MST: 50 nM RED fluorescence dye-labeled His6x-eIF4E in the absence (green) or presence of equimolar amounts of the 18aa N-terminal p20 peptide (yellow) with serially diluted m^7^GpppG-capped 64 nt long RNA (40 nt of SSA1 5′UTR), starting at 1 µM. Error bars indicate s.d. (n = 3).

To assess the importance of interaction between p20 and eIF4E for translation, we further tested a synthetic peptide consisting of the 18 N-terminal amino acids (aa) of p20 carrying the eIF4E-binding motif (peptide MIKYTIDELFQLKPSLTL; Supplemental Fig. S1a) in *in vitro* translation competition experiments as described above for other eIF4E-binding peptides. As opposed to full length p20 and comparable to the above tested eIF4E-binding peptides (Fig. 4), equimolar amounts of the N-terminal 18aa p20 peptide (1 pmol) nearly completely abolished the stimulatory effect of eIF4E on capped mRNA translation (Fig. 5a). Also similar to the other tested eIF4E-binding peptides, the 18aa p20 peptide had a greatly reduced effect on translation of capped mRNAs in the presence of eIF4E/p20 complex compared to eIF4E alone (even when added at a 5× excess; Fig. 5a). This provides further support for the stability of the eIF4E/p20 complex.

To gain additional insight into the mechanism by which the 18aa p20 peptide interferes with eIF4E-depedent translation, we assessed whether this peptide might act as an allosteric inhibitor of interaction between eIF4E and capped mRNA. We found, as indicated by MST experiments, that the affinity of eIF4E for capped RNA (K_d_ = 229.5 nM) is only slightly reduced in the presence of equimolar amounts of the 18aa p20 peptide (K_d_ = 210.7 nM) (Fig. 5b). This result indicates that the increased interaction of eIF4E/p20 complex with capped mRNA compared to that of eIF4E alone (K_d_ = 46.9 nM; Fig. 2a) is not solely due to interaction of eIF4E with the p20 N-terminal eIF4E-binding domain. This result is not unexpected, as capped mRNA and eIF4E-binding proteins are known to bind to very different domains on eIF4E^34^.

It would have been of interest to compare the binding affinity of eIF4E for the 18aa p20 peptide vs. full-length p20. Unfortunately, we could not compare the stability of such complexes since full-length recombinant p20 could not be efficiently purified from *E. coli*. We assume, however, that interaction of eIF4E with p20 is likely much stronger than with the 18aa p20 peptide, and that the canonical eIF4E-binding domain is necessary but not sufficient to explain the stability of the complex. Based on what has been reported for other eIF4E/4E-BP complexes of metazoan or plant origin (see Introduction), it is likely that other domains of p20 contribute to the stability of eIF4E/p20 complex.

## Discussion

In this paper, we describe enhanced binding of capped mRNAs by the yeast eIF4E/p20 complex as compared to eIF4E by itself. While eIF4E does not bind to uncapped mRNAs, we found that the eIF4E/p20 complex does, albeit with much lower affinity than for capped mRNAs. Our *in vitro* experiments showed dose-dependent enhancement of capped mRNA translation and inhibition of uncapped mRNA translation when either eIF4E or eIF4E/p20 complex were added to our eIF4E/p20-dependent cell-free translation lysate. The observed *in vitro* effects of exogenously added eIF4E or eIF4E/p20 on translation of capped and uncapped mRNAs were similar for all of reporter mRNAs used in this work, despite differences in the length and complexity of their 5′UTR sequences. Both enhancement of capped mRNA translation and inhibition of uncapped mRNA translation were stronger with addition of eIF4E/p20 complex compared to eIF4E alone, consistent with the observed increases in mRNA binding affinity in the presence of p20. The enhanced effects of purified eIF4E/p20 complex relative to eIF4E alone are likely due at least in part to the direct interaction of p20 with RNA identified in our work. We found that this involves a stretch of positively charged amino acids that does not affect p20-eIF4E binding and is similar to non-specific mRNA-binding sites in other proteins, including yeast eIF4G^35^. These positively charged regions presumably participate in electrostatic interactions with negatively charged phosphate residues in the mRNA^23^. The residues identified as important for p20-mRNA interaction have a similar location as one of the three mRNA-binding sites in eIF4G, lying about 50 to 100 amino acids downstream of the canonical eIF4E-binding motif^23^ (Supplemental Fig. S1b).

Thus, our data allow us to propose a novel mechanism whereby yeast eIF4E binding to capped mRNA is promoted by p20, resulting in a stable ternary complex. The combination of eIF4E binding to the cap-structure and p20 electrostatic interaction with adjacent nucleotides in the 5′UTR of capped mRNAs results in complexes that may act as a stable depository of capped mRNAs. The stability of the eIF4E/p20/mRNA complex is supported by our finding that inhibition of *in vitro* eIF4E-dependent translation of capped mRNA by equimolar amounts of 4E-binding peptides derived from eIF4G, Eap1 or p20 is much less pronounced when eIF4E is present in a complex with p20 rather than alone. We propose that the ternary eIF4E/p20/capped mRNA complex may exist in a dynamic equilibrium with other complexes comprised of yeast eIF4E and eIF4G or Eap1, and that various physiological conditions (e.g., nutrient deprivation, heat, drought and/or other forms of stress) might cause this equilibrium to shift towards translation initiation, mRNA degradation, and/or some other yet to be identified cellular processes involving mRNAs (e.g., nuclear transport as shown in mammalian cells^36^). Since p20 is a non-essential protein, the eIF4E/p20/mRNA ternary complex must not be essential either, at least under favorable physiological conditions. This could be different under stress conditions such as high temperature (37 °C or higher). Indeed, the p20 knockout yeast strain used in this work (RH2585 ∆p20) shows a temperature-sensitive phenotype (see Fig. 2d). It is possible that under natural (non-laboratory) conditions, survival in the face of common simultaneous stresses could depend upon mRNAs which would rely on p20 for translation.

Both eIF4E and p20 are rather abundant proteins, reported to be present in approximately equimolar amounts in the cell (~10,000–35,000 molecules/cell^37,38^). These protein levels correspond well with the estimated number of mRNAs in the cytoplasm of a yeast cell (~15,000–27,000 mRNA transcripts/cell^39–41^). Under physiological conditions, factors promoting either translation initiation (mediated by eIF4E-eIF4G interaction) or mRNA degradation (mediated by eIF4E-Eap1p interaction) could displace p20 from its complex with eIF4E and mRNA. However, both eIF4G and Eap1p have been determined to be present in lower amounts than p20 in yeast cells^37,38^. It is well accepted that eIF4G displaces p20 and other 4E-BPs to allow for efficient capped mRNA translation. As for the regulation of mRNA stability, while Eap1p has been shown to be involved in accelerated decay of specific capped mRNAs^42,43^, there is also clear evidence that p20 can influence mRNA decay. For example, p20 activity modulates phenotypes associated with the helicase Dhh1p and the RNA-binding protein Pat1p, which both interact with the 3′UTR of mRNAs slated for degradation and accelerate deadenylation-dependent decapping^44^. Thus, knockout of p20 alleviates the cold-sensitive phenotype of dhh1 knockout and overexpression of p20 inhibits growth of both ∆dhh1 and ∆pat1 yeast strains^6^. Additionally, our data suggests that the eIF4E-p20 complex may translationally silence uncapped mRNAs during the process of degradation (see Fig. 3b).

We anticipate that as for mammalian 4E-BPs, interaction of eIF4E and p20 may be regulated through posttranslational modifications such as phosphorylation of one or both proteins. However, while yeast p20 and eIF4E are both phosphoproteins, such modifications have not been shown to have an overall effect on translation to date^20^. Thus, further investigation into the mechanisms controlling p20 protein levels and binding capacity for eIF4E and mRNA is warranted.

In summary, our study proposes a more complex role of p20 function in yeast cells, acting not only as a general repressor of translation, but rather as both negative and positive regulator of protein synthesis.

## Methods

### Protein expression and purification

DNA sequences encoding codon-optimized N-terminally His6x-tagged eIF4E (plasmid His6x-eIF4E-pet3a) or p20 (plasmid His6x-p20-pet3a) were cloned into pET3a vector digested with NdeI and BamHI. For co-expression of both proteins, a plasmid directing expression of untagged eIF4E was generated via PCR amplification using plasmid His6x-eIF4E-pet3a as a template. The resulting DNA fragment with a NcoI restriction site at its 5′end followed by the entire open reading frame of eIF4E and a BamHI restriction site was ligated into NcoI-BamHI double-restricted pet28a vector to yield plasmid eIF4E-pet28a. p20 mutants R55-57L (3×) and R55-57L + H60-62L (6×) were obtained by site-directed mutagenesis (QuikChange Site-Directed Mutagenesis Kit, Agilent). Competent *E. coli* BL21 Rosetta 2 (DE3) cells (Novagen) were transformed either with plasmid His6x-eIF4E-pet3a (for expression of eIF4E alone) or with plasmids His6x-p20-pet3a (wild type or mutant) and eIF4E-pet28a (for expression of eIF4E/p20 complex).

eIF4E and eIF4E/p20 were expressed in *E. coli* BL21 Rosetta 2 (DE3) cells grown in LB medium. Expression was induced with 0.5 mM IPTG overnight at 18 °C. Cells were harvested by centrifugation (3000 × g, 6 min, 4 °C) and the pellet was suspended in lysis buffer (50 mM Tris.HCl pH 8, 500 mM NaCl, 20% Glycerol, 1% Tween 20, 10 mM Imidazole, 20 mM beta-Mercaptoethanol) supplemented with DNaseI (5 mg/mL), lysozyme (1 mg/mL) and Complete Protease Inhibitor Cocktail (Roche). The cell suspension was incubated for 20 min on ice and sonicated at 50% amplitude for 30 s on/off for several cycles. Cell debris was removed by ultracentrifugation (120,000 × g, 30 min, 4 °C) and the resulting lysate was then incubated with Ni-NTA Agarose (Invitrogen) for 60 min at 4 °C with gentle rotation. After washing the resin three times with wash buffer (50 mM Tris.HCl pH 8, 500 mM NaCl, 20 mM Imidazole, 10 mM beta-Mercaptoethanol), bound protein was eluted by incubation with elution buffer (50 mM Tris.HCl pH 8, 500 mM NaCl, 250 mM Imidazole, 10 mM beta-Mercaptoethanol) for 30 min at 4 °C with gentle rotation.

Yeast eIF4G peptides aa160-492, aa441-492, mutant LL459,460/AA and Eap1 peptide aa106-273 were expressed as GST-fusions from pGEX1 or pGEX3 vectors in *E. coli* BL21 Rosetta 2 (DE3) cells grown in LB medium. Expression was induced with 0.5 mM IPTG for 2 h at 37 °C. Site-directed mutagenesis of the pGEX1 eIF4G aa160-492 plasmid was performed by PCR with oligonucleotides designed to simultaneously introduce mutations L459A (TTG to GCG) and L460A (CTT to GCT) to obtain mutant LL459,460/AA. GST fusion proteins were purified as described previously^4^.

All purified proteins were concentrated using Amicon Ultra-2 Centrifugal Filters (Merck) and dialyzed with Slide-A-Lyzer MINI Dialysis Devices (Thermo Scientific) in either buffer A (10 mM Sodiumphosphate pH 7.5, 100 mM NaCl, 2.5 mM MgCl2, 1 mM DTT, 0.05% NP40) for microscale thermophoresis assay (MST) and electrophoretic mobility shift assay (EMSA) or buffer B (20 mM HEPES.KOH pH 7.5, 50 mM NaCl, 1 mM DTT) for *in vitro* translation assay.

The N-terminal 18aa p20 peptide (MIKYTIDELFQLKPSLTL; molecular weight: 2153.59 g/mol, HPLC purity 98.9%) was synthesized by GenScript USA, Inc. and confirmed to have proper water solubility at a concentration of 5 mg/mL.

### Synthesis of mRNA transcripts for use in *in vitro* translation assays

The 5′UTRs of GRE1 (79 nt), PMA1 (244 nt) and SSA1 (40 nt) were obtained by PCR amplification from yeast genomic DNA. Due to the presence of restriction enzyme sites in the PCR primers, the resulting DNA fragments contained a SmaI restriction site at the 5′ end followed by the 5′UTR sequence and an NcoI restriction site at the 3′ end. PCR-generated fragments were digested with SmaI and NcoI and then cloned into the 5′UTR of the pRL Renilla Luciferase Reporter vector (see Supplemental Fig. S1e).

The pRL-based constructs containing GRE1, PMA1 or SSA1 5′UTRs were linearized with XhoI to produce “run off” transcripts consisting of the inserted 5′UTR sequence (GRE1, PMA1 and SSA1) followed by the Renilla Luciferase ORF and a poly(A) tail. Synthesis of capped or uncapped mRNAs from these constructs was accomplished using T3 RNA Polymerase (Promega) according to the manufacturer’s instructions. At the end of the transcription reaction, RQ1 RNase-free DNase (Promega) was added to a concentration of 1 U/μg template DNA and incubated for 1 h at 37 °C to digest the DNA template. RNA was extracted with citrate-saturated phenol:chloroform:isoamyl alcohol (125:24:1, pH 4.7), followed by chloroform:isoamyl alcohol (24:1) and then precipitated using sodium acetate*/*ethanol. ∆G was obtained using http://kinefold.curie.fr/.

### mRNA synthesis for MST and EMSA

To obtain short uncapped RNAs (64 nt) for MST or EMSA, the pRL Renilla Luciferase Reporter vector containing the SSA1 5′UTR (40 nt) was linearized with NcoI to produce a “run off” transcript. RNA synthesis with T3 RNA Polymerase (Promega) and purification was performed as described above. Vaccinia Capping System (NEB) was used according to the manufacturer’s recommendations to add the 7-methylguanylate cap-structure to the 5′end of RNAs. Capped RNAs were purified by phenol-chloroform extraction and gel filtration (Sephadex G-50, Sigma). Successful capping evidenced by a shift in mass was verified by electrophoresis using 20% polyacrylamide gels containing 8 M urea, stained with 2 g/L methylene blue in 0.4 M NaAc, pH 4.7 and destained in water.

### Microscale thermophoresis assay (MST)

All microscale thermophoresis measurements were performed at 25 °C using Monolith NT™ standard capillaries and the Monolith NT.115 device (NanoTemper Technologies GmbH). Experiments were repeated three times for each measurement. Data analyses were carried out using NanoTemper MO.Affinity Analysis software. The affinity between purified eIF4E or eIF4E/p20 complex and synthetic 40 nt uncapped 3′FAM labeled RNAs (Microsynth, Switzerland) was measured at 30% LED and 40% MST power with Laser-On time 30 s and Laser-Off time 5 s. Fluorescently labeled nucleic acid samples were diluted in buffer A to 50 nM. For each binding analysis, a titration series was prepared with varying concentrations of eIF4E or eIF4E/p20 complex in buffer A.

For MST experiments with *in vitro* transcribed capped RNA, purified eIF4E protein and purified eIF4E/p20 complex were labeled with the Monolith His-Tag Labeling Kit RED-tris-NTA (NanoTemper Technologies GmbH) in buffer B. Measurements were performed with varying concentrations of RNA in buffer A and 50 nM of labeled eIF4E or eIF4E/p20 complex at 40% LED and 40% MST power with Laser-On time 30 s and Laser-Off time 5 s.

### Electrophoretic mobility shift assay (EMSA)

A standard reaction (20 µL in buffer A) consisted of 1 µM synthetic 40 nt uncapped 3′FAM labeled RNA (Microsynth, Switzerland) or 0.25 µM *in vitro* transcribed capped RNA and varying concentrations of eIF4E or eIF4E/p20 complex. The RNA was heated to 60 °C and allowed to cool to room temperature before use. Immediately prior to loading of binding reactions onto gels, 5 µL loading buffer (30% (v/v) glycerol, 0.25% (w/v) bromphenol blue) was added to each reaction.

Binding reactions were run on 1.5% agarose gels in 1× TBE buffer for 40 min at 80 V. RNAs were visualized by SYBR Gold Nucleic Acid Gel Stain (Invitrogen) or by the fluorescent label (3′Fluorescein, FAM) using a Fusion FX SkyLight transilluminator (Vilber Lourmat, France). The fluorescence intensity of unbound RNA was determined as a function of protein concentration using Fusion FX software and the dissociation constant (K_d_) was calculated using GraphPad Prism7 (nonlinear regression, one site specific binding).

### *In vitro* translation assay

Preparation of extracts from eIF4E ts4-2 mutant and p20 knockout yeast strains was performed as described previously^32^ with some modifications. 250 mL yeast culture was grown overnight in YPD medium at 30 °C to a final OD_600_ of 2. Cells were harvested, resuspended in buffer ADPM (30 mM HEPES.KOH pH 7.4, 100 mM KOAc, 2 mM Mg(OAc)_2_, 2 mM DTT, 0.1 mM PMSF, 8% mannitol), and pipetted into liquid nitrogen to form small pellets. Cells were disrupted by cryogenic grinding and the resulting paste was centrifuged at 15000 × g for 10 min at 4 °C. The supernatant was recovered and loaded onto a Sephadex G25 (fine) column equilibrated with buffer ADP (ADPM without mannitol) in order to remove low-molecular-weight components. Fractions were collected, the OD_260_ was measured and main fractions (OD_260_ > 100) were pooled and frozen (−75 °C) in aliquots.

For *in vitro* translation reactions, the following components were incubated for 60 min at 25 °C: 1.5 µL extract, 1.04 µL 6xAA cocktail (132 nM HEPES.KOH pH 7.4, 720 mM K-Acetate, 9 mM Mg-Acetate, 4.5 mM ATP, 0.6 mM GTP, 150 mM Creatine Phosphate, 0.24 mM 20 amino acid mixture, 10.2 mM DTT), 0.15 µL RNasin Ribonuclease Inhibitor 40 U/µL (Promega), 10 ng mRNA, 1 µL protein in ADP buffer. *In vitro* synthesized products were quantitated with the Renilla Luciferase Assay System (Promega) and a GloMax 20/20 Luminometer (Promega). Measurements were obtained from three independent experiments.

### *In vivo* analyses

For *in vivo* analyses, yeast strain RH2585 (*MAT*α *his3::hisG trp1::hisG ura3-52*, derivative of Σ1278b) carrying a deletion in the gene encoding p20 (*caf20::NatR*) or isogenic yeast strains with integrated plasmids directing expression of p20 wild-type, R55-57L (3×) or R55-57L, H60-62L (6×) mutants were spotted on YPD medium in serial 10-fold dilutions starting with 10,000 cells/μL. Plates were photographed after incubation at 30° or 37 °C for 3 days. Samples (½ OD_600_) of the same yeasts were collected from liquid cultures at midlog growth phase (30° or 37 °C) and loaded directly onto SDS-PAGE gels. Total cell extracts were prepared from liquid cultures at midlog growth phase (30° or 37 °C) harvested at OD_600_ = 20. Yeast cells were washed once with and resuspended in buffer ADP (30 mM Hepes.KOH pH 7.4, 100 mM KOAc, 2 mM Mg(OAc)_2_, 2 mM DTT, 0.1 mM PMSF) then lysed by vortexing with glass beads. m^7^GDP-Sepharose was washed twice with buffer ADP and 300 µg of total extract was then added to the resin and incubated at 4 °C for 1 hour on a rotating wheel. The supernatant containing unbound proteins was removed and the resin was washed three times with buffer ADP then incubated for 15 min at 4 °C with 1 mM m^7^GDP (in ADP) to elute bound proteins. 10 µg of extract or the complete eluate from each binding reaction was boiled in 2× SDS sample buffer and loaded onto a 17.5% SDS-PAGE gel. For Western blotting, proteins were transferred from the gel to a nitrocellulose membrane (BioRad) which was stained first with polyclonal rat anti-eIF4E antibody (1:1000 dilution) followed by polyclonal rabbit anti-rat IgG-HRP (1:3000 dilution; Dako, Denmark), and then by polyclonal rat anti-p20 (dilution) followed by polyclonal rabbit anti-rat IgG-HRP. The primary antibodies were raised against full-length p20 and yeast eIF4E expressed and purified from *E. coli*. Protein signals were detected using WesternBright^TM^ ECL (Advansta, USA) and FusionFX (Vilber Lourmat, France) reagents. Signals strengths were calculated using ImageJ software (https://imagej.nih.gov/ij/).

### Data availability statement

There are no restrictions to availability of materials and data presented in this work. All requests for information and materials should be sent to MA (michael.altmann@ibmm.unibe.ch).

## Electronic supplementary material

Supplementary Information
