# Supplementary material for: Properties of the ternary complex formed by yeast eIF4E, p20 and mRNA
Source: Sci Rep. 2018 Apr 30;8:6707. doi: 10.1038/s41598-018-25273-3 (PMC5928113; doi:10.1038/s41598-018-25273-3)
Supplement: Supplementary file 1 — Supplementary Information [file 41598_2018_25273_MOESM1_ESM.pdf]

Supplementary Information

Properties of the ternary complex formed by yeast eIF4E, p20 and mRNA

Nick Arndt[1], Daniela Ross-Kaschitza[1], Artyom Kojukhov[2], Anton A. Komar[2] and Michael Altmann[1*]

[1]Institut für Biochemie und Molekulare Medizin, Bühlstr. 28, 3012 Bern, Switzerland

[2]Center for Gene Regulation in Health and Disease and Department of Biological, Geological and Environmental Sciences, Cleveland State University, Cleveland, OH 44115, USA

*Corresponding author: michael.altmann@ibmm.unibe.ch

Supp. Fig.1:

S1a) Amino acid sequence of yeast p20 (P12962) showing the canonical eIF4E-binding site (aa 4-10; marked in green) and arginine (aa55-57) or histidine residues (aa60-62) that were mutated to leucine in this work (marked in cyan). The N-terminal 18aa p20 peptide used in this work is underlined.

```
  1 MIKYTIDELF QLKPSLTLEV NFDAVEFRAI IEKVKQLQHL KEEEFNSHHV GHFGRRRSSH
 61 HHGRPKIKHN KPKVTTDSDG WCTFEAKKKG SGEDDEEETE TTPTSTVPVA TIAQETLKVK
121 PNNKNISSNR PADTRDIVAD KPILGFNAFA ALESEDEDDE A*
```

S1b) Schematic depiction of yeast eIF4G, Eap1 and p20 proteins. The canonical YxxxxLL/Y/F eIF4E-binding motif found in each protein is given on the right and its position is indicated by a vertical purple bar. The amino acids corresponding to the eIF4G and Eap1 peptides used in this study are shown as grey horizontal lines.

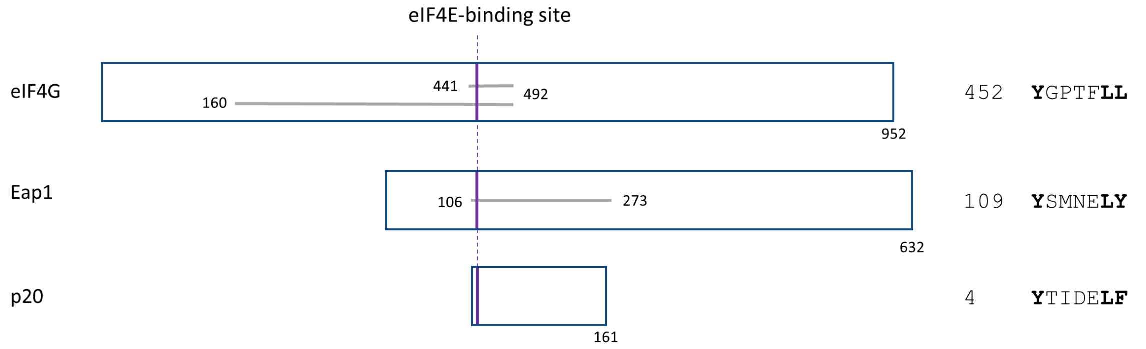

S1c) Sequence comparison of yeast eIF4E native sequence (top line) vs. codon optimized seqeunce (DAPCEL; bottom line)

```
YEAST_eIF4E       ---ATGTCCGTTGAAGAAGTTAGCAAGAAGTTTGAAGAAAACGTTTCAGTCGATGATACC
eIF4E_DAPCEL      ATGGGCAGCGTTGAAGAAGTCTCCAAAAAATTTGAAGAAAACGTTAGCGTAGATGATACT
                          ************   *** ** ***************   ** ********

YEAST_eIF4E       ACAGCTACTCCAAAGACTGTTTTAAGTGACAGTGCTCACTTCGATGTCAAGCACCCATTG
eIF4E_DAPCEL      ACCGCGACCCCGAAAACCGTTTTAAGCGACAGCGCGCACTTCGATGTAAAACACCCGTTA
                  ** ** ** ** ** ** ******** ***** ** *********** ** ***** **

YEAST_eIF4E       AACACCAAATGGACTTTATGGTACACAAAGCCAGCCGTCGATAAATCTGAGTCGTGGTCT
eIF4E_DAPCEL      AATACTAAATGGACCTTATGGTACACCAAACCGGCTGTAGATAAAAGCGAGTCGTGGAGC
                  ** ** ******** *********** ** ** ** ** ******   *********

YEAST_eIF4E       GATCTATTACGTCCCGTCACTTCATTCCAAACTGTTGAAGAATTTTGGGCTATCATTCAA
eIF4E_DAPCEL      GATTTGTTAAGACCCGTAACCAGCTTCCAGACCGTTGAAGAATTTTGGGCGATCATTCAG
                  *** * *** * ***** **    ***** ** ***************** ********

YEAST_eIF4E       AATATTCCTGAGCCACACGAACTACCATTGAAATCAGATTACCACGTCTTCCGTAATGAC
eIF4E_DAPCEL      AATATTCCGGAGCCGCACGAATTGCCGTTAAAAAGCGATTACCACGTATTCAGAAATGAC
                  ******** ***** ****** * ** ** ***   *********** *** * ******

YEAST_eIF4E       GTTAGACCTGAATGGGAAGATGAAGCCAATGCTAAAGGTGGTAAATGGTCTTTCCAACTT
eIF4E_DAPCEL      GTTCGTCCTGAATGGGAAGATGAAGCTAATGCGAAAGGTGGTAAATGGAGCTTCCAGTTG
                  *** * ******************** ***** ***************   *****  *

YEAST_eIF4E       AGAGGAAAAGGTGCTGATATTGATGAATTATGGCTAAGAACTTTACTAGCAGTTATTGGT
eIF4E_DAPCEL      CGTGGGAAAGGTGCGGATATTGATGAATTATGGTTGCGTACCTTATTGGCTGTTATTGGT
                   * ** ******** ****************** *  * ** *** * ** *********
```

```
YEAST_eIF4E       GAAACAATTGATGAAGACGACTCCCAAATTAACGGTGTCGTTTTAAGCATTAGAAAAGGT
eIF4E_DAPCEL      GAAACCATTGATGAAGACGACAGCCAGATTAACGGTGTAGTTTTATCCATTCGTAAAGGT
                  ***** ***************  *** *********** ******  **** * ******

YEAST_eIF4E       GGTAACAAGTTTGCCTTATGGACTAAATCTGAAGACAAAGAACCACTATTGAGAATTGGT
eIF4E_DAPCEL      GGTAATAAATTTGCTTTATGGACCAAAAGCGAAGACAAAGAACCGTTGTTACGTATTGGT
                  ***** ** ***** ******** ***   **************  * **  * ******

YEAST_eIF4E       GGTAAATTCAAGCAAGTTTTAAAATTAACCGATGACGGGCATTTGGAATTCTTTCCACAT
eIF4E_DAPCEL      GGTAAATTCAAACAGGTTTTAAAATTAACTGATGACGGGCATTTAGAATTTTTTCCGCAT
                  *********** ** ************** ************** ***** ***** ***

YEAST_eIF4E       TCCAGTGCCAATGGTAGACACCCTCAACCATCAATCACCTTGTAA
eIF4E_DAPCEL      AGCAGCGCTAATGGTCGTCACCCGCAGCCGAGCATCACTTTATAA
                    *** ** ****** * ***** ** **    ***** ** ***
```

S1d) Sequence comparison of yeast p20 native sequence (top line) vs. codon optimized sequence (DAPCEL; bottom line)

```
YEAST_p20       ATGATCAAGTATACTATCGATGAGCTTTTTCAACTGAAGCCAAGTTTAACTTTGGAAGTT
p20_DAPCEL      ATGATCAAATATACCATCGATGAGTTGTTTCAGCTCAAACCGAGCTTAACTTTAGAAGTT
                ******** ***** ********* * ***** ** ** ** ** ******** ******

YEAST_p20       AATTTCGATGCGGTGGAATTTAGAGCCATCATTGAAAAAGTTAAGCAATTGCAACACTTG
p20_DAPCEL      AATTTCGATGCTGTAGAATTTCGTGCTATCATCGAAAAAGTTAAACAGTTACAGCACTTA
                *********** ** ****** * ** ***** *********** ** ** ** *****

YEAST_p20       AAAGAGGAAGAGTTTAACAGTCATCATGTTGGTCATTTCGGTCGTAGAAGATCTTCCCAC
p20_DAPCEL      AAAGAGGAAGAGTTTAACAGCCATCATGTTGGTCATTTCGGTAGACGTCGTAGCTCCCAC
                ******************** ********************* *  *  *    ******

YEAST_p20       CATCATGGTAGACCAAAGATTAAGCACAACAAGCCTAAGGTTACAACCGATTCAGATGGT
p20_DAPCEL      CATCATGGTCGTCCGAAAATTAAACACAATAAACCGAAAGTTACCACTGATAGCGATGGT
                ********* * ** ** ***** ***** ** ** ** ***** ** ***   ******


YEAST_p20       TGGTGCACATTTGAAGCCAAGAAGAAGGGTAGTGGAGAAGATGATGAAGAAGAAACAGAA
p20_DAPCEL      TGGTGCACCTTTGAAGCTAAAAAAAAAGGTAGCGGGGAAGATGATGAAGAAGAAACCGAA
                ******** ******** ** ** ** ***** ** ******************** ***

YEAST_p20       ACCACACCAACTTCTACTGTGCCAGTTGCTACCATTGCCCAAGAAACTTTAAAAGTCAAG
p20_DAPCEL      ACTACCCCGACCAGCACCGTACCGGTTGCGACTATTGCTCAGGAAACCTTAAAAGTAAAA
                ** ** ** **    ** ** ** ***** ** ***** ** ***** ******** **

YEAST_p20       CCAAATAACAAAAATATTTCTTCCAACAGACCTGCTGATACCAGAGATATTGTTGCGGAC
p20_DAPCEL      CCGAATAACAAAAATATCTCTTCCAACCGCCCCGCGGATACTCGTGATATTGTTGCTGAC
                ** ************** ********* * ** ** *****  * *********** ***

YEAST_p20       AAGCCAATTCTTGGTTTCAACGCATTTGCTGCTTTGGAAAGTGAAGACGAAGACGACGAA
p20_DAPCEL      AAACCGATTTTGGGTTTCAATGCTTTTGCGGCGTTAGAAAGCGAAGACGAAGACGACGAA
                ** ** *** * ******** ** ***** ** ** ***** ******************

YEAST_p20       GCATAA
p20_DAPCEL      GCTTGA
                ** * *
```

S1e) Nucleotide sequence of RNAs used in this work:

3'FAM labeled 40 nt SSA1 RNA (Microsynth AG), used for MicroScale Thermophoresis™ or Electrophoretic Mobility Shift Assay measurements	GUAUUACAAGAAACAAAAAUUCAAGUAAAUAACAG AUAAU
In vitro transcribed, m^7GTP- or m^7GpppG-capped 64 nt SSA1 5'UTR RNA, used for MicroScale Thermophoresis™ or Electrophoretic Mobility Shift Assay measurements	GGGAACAAAAGCUGGUAAUCAAGAAUUACAAGAAA CAAAAAUUCAAGUAAAUAACAGAUAAUAC
"Average" 5'UTR fused to Renilla Luciferase ORF (AUG marked in bold), used for *in vitro* translation assays	GGGAACAAAAGCUGGAGCUCGCCCGGGCUGUUCUA GCCACC**AUG**G

PMA1 5'UTR fused to Renilla Luciferase ORF (AUG marked in bold), used for *in vitro* translation assays	GGGAACAAAAGCUGGAGCUCGCCCGGGACACCAAU<br>AGUGAAAAUCUUUUUUUCUUCUAUAUCUACAAAAA<br>CUUUUUUUUUCUACAAAAACUUUUUUUUUCUAUCA<br>ACCUCGUUGAUAAAUUUUUUCUUUAACAAUCGUUA<br>AUAAUUAAUUAAUUGGAAAAUAACCAUUUUUUCUC<br>UCUUUUAUACACACAUUCAAAAGAAAGAAAAAAAA<br>UAUACCCCAGCUAGUUAAAGAAAAUCAUUGAAAAG<br>AAUAAGAAGAUAAGAAAGAUUUAAUUAUCAAACAA<br>UAUCAAUCC**AUG**G
GRE1 5'UTR fused to Renilla Luciferase ORF (AUG marked in bold), used for *in vitro* translation assays	GGGAACAAAAGCUGGAGCUCGCCCGGGGAGCCAAA<br>CACUACCGCAUAAAAGCUAAGUACGAAUAACAAUU<br>AAGAACC**AUG**G
SSA1 5'UTR fused to Renilla Luciferase ORF (AUG marked in bold), used for *in vitro* translation assays	GGGAACAAAAGCUGGUAAUCAAGAAUUACAAGAAA<br>CAAAAAUUCAAGUAAAUAACAGAUAAUACC**AUG**G

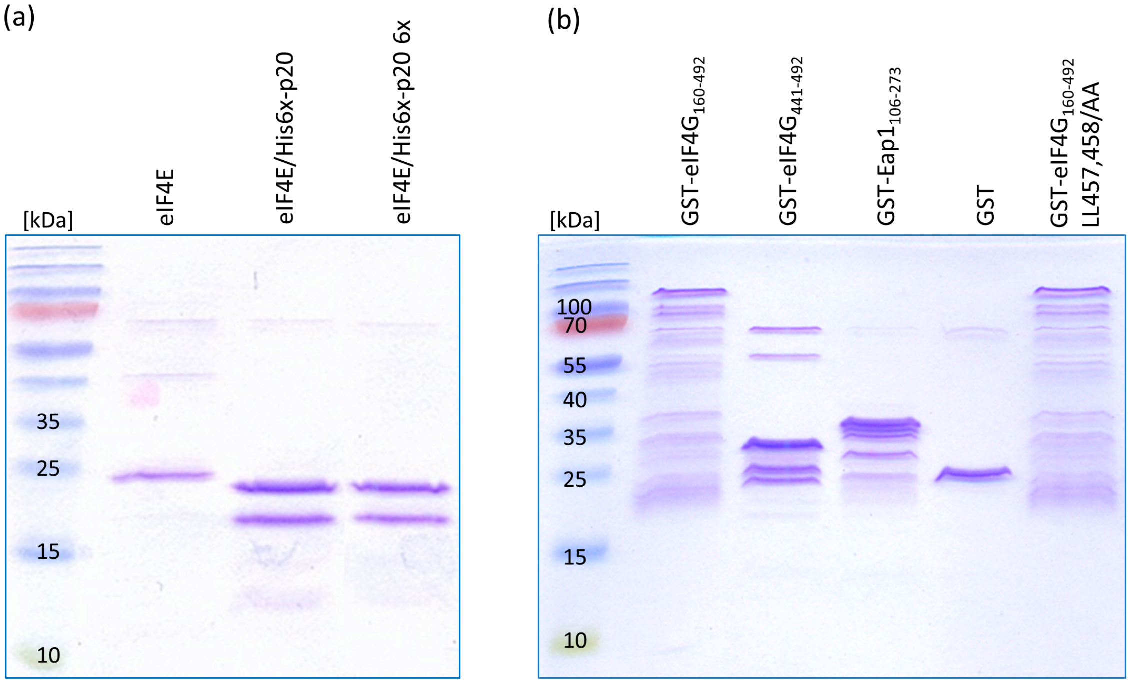

Supp. Fig.2: Coomassie stained SDS-PAGE (20%)

(S2a) 0.75 µg purified His6x-eIF4E alone (lane 1), 2.0 µg co-expressed and purified eIF4E/His6x-p20 complex (lane 2), and 2.0 µg co-expressed and purified eIF4E/His6x-p20 6x mutant complex (lane 3)

(S2b) 4 µg GST-eIF4G$_{160\text{-}492}$ (lane 1), 4 µg GST-eIF4G$_{441\text{-}492}$ (lane 2), 4 µg GST-Eap1$_{106\text{-}273}$ (lane 3), 4 µg GST (lane 4), and 4 µg GST-eIF4G$_{160\text{-}492}$ LL457,458/AA mutant (lane 5).

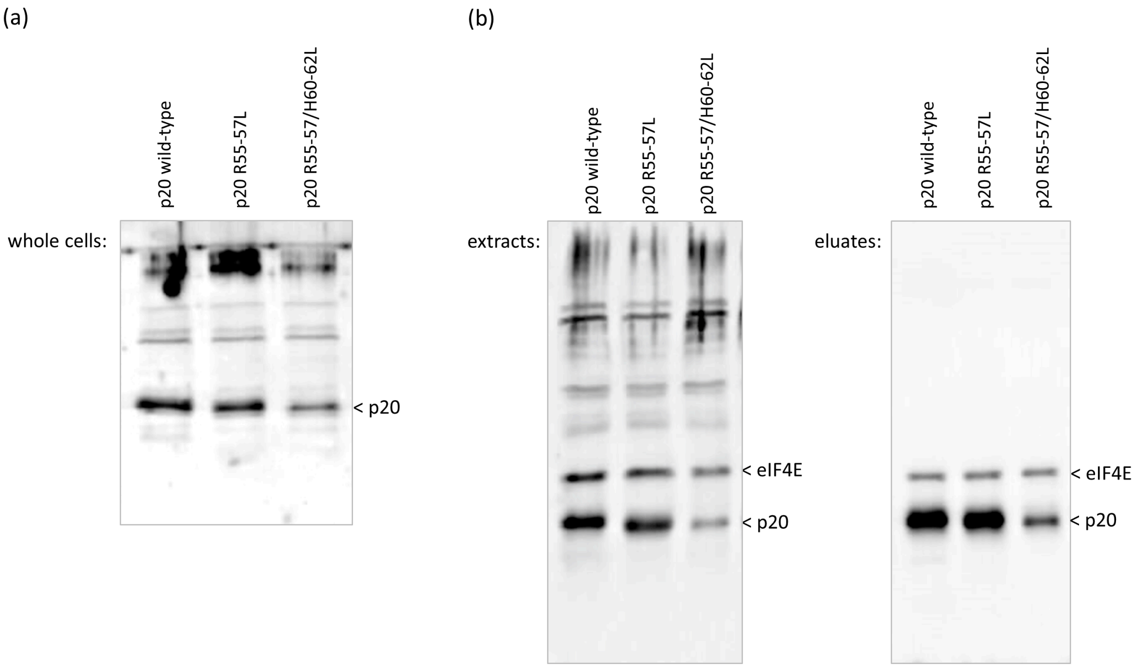

Supp. Fig. 3: Full-length Western blots (sections of which are presented in Figure 2d). Blots were stained with polyclonal antibodies against eIF4E or p20 as described in Methods.

S3a) Western blot of ½ OD_{600} whole yeast cells carrying p20 knockout (RH2585 Δp20) and expressing p20 wild type, R55-57L (3x) or R55-57L, H60-62L (6x) mutants.

S3b) Western blots of input extracts (10 µg loaded on blot) used for m^7GDP-Sepharose pulldown (left panel) and eluates (right panel) for p20 wild type, R55-57L (3x) and R55-57L, H60-62L (6x) mutants.
